# Impact of Ertugliflozin on Cardiac Structure and Function in Patients with ICDs/CRT-Ds Assessed by Echocardiography: A Post Hoc Sub-Analysis of the ERASe Trial

**DOI:** 10.3390/jcm14238294

**Published:** 2025-11-22

**Authors:** Viktoria Santner, Ewald Kolesnik, Martin Benedikt, Abderrahim Oulhaj, Ursula Rohrer, Martin Manninger, Norbert J. Tripolt, Peter N. Pferschy, Faisal Aziz, Markus Wallner, Nora Schwegel, Nicolas Verheyen, Klemens Ablasser, Johannes Gollmer, Marianne Gwechenberger, Martin Martinek, Michael Nürnberg, Clemens Steinwender, Andreas Zirlik, Markus Stühlinger, Harald Sourij, Dirk von Lewinski, Daniel Scherr

**Affiliations:** 1 Division of Cardiology, University Heart Center and Department of Internal Medicine, Medical University of Graz, 8010 Graz, Austria; 2 Department of Public Health and Epidemiology, College of Medicine and Health Sciences, Khalifa University of Sciences and Technology, Abu Dhabi P.O. Box 17666, United Arab Emirates; 3 Division of Endocrinology and Diabetology, Department of Internal Medicine, Medical University of Graz, Auenbruggerplatz 15, 8036 Graz, Austria; 4 Cardiometabolic Trials Unit, Medical University of Graz, 8036 Graz, Austria; 5 Department of Cardiology, Medical University of Vienna, 1090 Vienna, Austria; 6 Internal Medicine II with Cardiology, Angiology and Intensive Care Medicine, Ordensklinikum Linz Elisabethinen, 4020 Linz, Austria; 7 3rd Medical Department with Cardiology and Intensive Care Medicine, Klinik Ottakring, 1160 Wien, Austria; 8 Department of Cardiology, Kepler University Linz, Medical Faculty, Kepler University Hospital Linz, 4020 Linz, Austria; 9 University Clinic of Internal Medicine III/Cardiology and Angiology, Medical University of Innsbruck, 6020 Innsbruck, Austria

**Keywords:** echocardiography, SGLT2 inhibitors, ertugliflozin, arrhythmia, ICD, CRT, heart failure, clinical trial

## Abstract

**Background/Objectives**: Ertugliflozin showed a beneficial effect to reduce arrhythmic burden in heart failure patients with an ejection fraction less than 50% with an implantable cardioverter-defibrillator (ICD) with or without cardiac resynchronization therapy device (CRT-D) in the ERASe trial. The aim of the post hoc sub-analysis of the ERASe trial was to compare the effect of ertugliflozin versus placebo on structural and functional parameters assessed by echocardiography. **Methods**: In this sub-analysis of the ERASe trial, 40 patients (87% of the overall ERASe trial cohort) with stored echocardiography loops and adequate image quality were included. A post hoc analysis including structural and functional echocardiography parameters was performed by a trained investigator blinded to the treatment group. **Results**: After 52 weeks of treatment, there were no between-group differences in left and right ventricular dimensions (left ventricular end-diastolic and -systolic volumes, right ventricular diameter) and function (left ventricular ejection fraction [LVEF], tricuspid annular plane systolic excursion), after adjustment for baseline values. Lower LVEF at baseline was associated with a higher number of ventricular arrhythmias in both treatment groups. **Conclusions**: In this sub-analysis of the ERASe trial, treatment with ertugliflozin did not improve structural and functional parameters assessed by echocardiography after 52 weeks of treatment compared to placebo despite the significant reduction in arrhythmic burden.

## 1. Introduction

Sodium–glucose co-transporter 2 inhibitors (SGLT2is) have established their role as the fourth pillar of heart failure (HF) therapy over the last few years and became a class IA treatment indication in all HF patients, regardless of left ventricular ejection fraction (LVEF), in 2023 [1,2]. There is strong evidence from large randomized controlled trials (RCT) showing the beneficial effects of dapagliflozin and empagliflozin on cardiovascular outcomes in HF patients [3,4,5,6]. Evidence showing the impact of SGLT2is on arrhythmic burden has been lacking. A post hoc analysis of the DAPA-HF trial showed a reduction in ventricular arrhythmias and sudden death in HF patients with reduced ejection fraction treated with dapagliflozin on top of standard HF medication [7]. The ERASe trial was an RCT evaluating the effect of the SGLT2i ertugliflozin on ventricular arrhythmias in patients with HF and an LVEF of less than 50% with an implantable cardioverter-defibrillator (ICD) with or without cardiac resynchronization therapy device (CRT-D) [8]. Ertugliflozin showed a significant reduction in arrhythmic burden after 52 weeks of treatment with an event rate of the primary endpoint of 3.5 (95% confidence interval [CI] 2.8, 4.4) in the ertugliflozin group compared with 13.3 in the placebo group (CI 11.8, 14.8, *p* < 0.001) [2,9]. The underlying pathways leading to an increased arrhythmic burden in patients with HF and impaired LVEF are not fully understood. Adverse cardiac remodeling and myocardial fibrosis seem to play an important role as arrhythmogenic substrates in those patients [10]. There is evidence that SGLT2is have an indirect antifibrotic effect [11]. The EMpaglifozin in MYocardial infarction (EMMY) trial underlines the potential effect of SGLT2is on cardiac remodeling, showing a significant effect on left ventricular volumes after treatment with empagliflozin for 26 weeks after myocardial infarction compared to placebo in the post hoc echocardiography sub-analysis [12,13].

This post hoc sub-analysis of the ERASe trial aimed to investigate whether treatment with ertugliflozin compared to placebo improved cardiac remodeling, assessed by echocardiographic structural and functional parameters.

## 2. Materials and Methods

### 2.1. The ERASe Trial

The ERASe Trial was a multicenter, double-blind, randomized, placebo-controlled trial to investigate the impact of the SGLT2i ertugliflozin on arrhythmic burden in HF patients with non-preserved ejection fraction (<50%). Patients had to have an ICD with or without CRT-D for more than three months and suffered from at least 10 episodes of ventricular tachycardia (including non-sustained ventricular tachycardia [nsVT] or sustained ventricular tachycardia [sVT]) within the last 12 months with or without ICD therapy. Patients were randomized 1:1 to receive either 5 mg of ertugliflozin once daily or placebo for 52 weeks. Primary endpoint of the study was the difference in number of sVT or ventricular fibrillation (VF) episodes after 52 weeks between treatment groups. Secondary endpoints included the number of nsVT, appropriate ICD therapies, changes in N-terminal pro-hormone of brain natriuretic peptide (NT-proBNP), and the rate of hospital re-admissions for HF. The ERASe trial was approved by the Ethics committee of the Medical University of Graz, Austria (EK 32-492 ex 19/20, EudraCT 2020-002581-14) and registered at ClinicalTrials.gov (NCT04600921). A detailed description of the study design, eligibility criteria, baseline characteristics, and trial results have been previously published [8,9]. As the updated 2023 ESC guidelines recommended the treatment with SGLT2i as a class 1A indication in all HF patients across the whole LVEF spectrum, the trial had to be terminated prematurely due to ethical considerations [2]. In a final cohort of 46 patients (11% of the calculated sample size), ertugliflozin compared to placebo reduced ventricular arrhythmias significantly by 80%. However, the results should be interpreted with caution as the trial was underpowered due to the limited sample size [9].

### 2.2. The Post Hoc Echocardiography Sub-Analysis

In the post hoc sub-analysis of the ERASe trial, analyses from stored echocardiographic loops of four participating centers were performed by a trained investigator blinded to study drug randomization (EK) using the software Ultrasound Workspace Version TTA2.53, TomTec Imaging Systems GmbH, Munich, Germany. Two-dimensional parameters including chamber quantification were assessed according to international recommendations and guidelines [14,15,16,17,18,19]. LVEF was calculated using Simpson’s Biplane method. M-mode was used to assess tricuspid annular plane systolic excursion (TAPSE). Myocardial deformation analysis resulting in left ventricular global longitudinal strain (LV GLS) was assessed using the automatically generated auto-strain with, if needed, manual readjustment of contours to ensure accurate tracking [20]. All analyses were conducted only if adequate image quality allowed it to, at the discretion of the blinded investigator.

### 2.3. Statistical Analysis

Categorical variables were reported as absolute frequencies (%) and continuous variables as means ± standard deviation. Baseline characteristics were compared between treatment groups using the chi-square or Fischer exact test, or unpaired *t*-tests, as appropriate. Differences in echocardiographic parameters between treatment groups after 52 weeks were assessed with a linear regression model after adjustment for baseline values. Associations between echocardiographic parameters at baseline with occurrence of ventricular arrhythmias and their interaction with treatment groups were estimated using negative binomial regression adjusting for baseline values of arrythmias and treatment. All statistical analyses were performed with R version 4.5.1. Results were reported with a confidence interval of 95%, and a *p*-value < 0.05 was considered to be statistically significant.

## 3. Results

### 3.1. Baseline Characteristics of the Overall Cohort

From the 46 patients included in the final analysis of the ERASe trial, 6 patients had to be excluded from the echocardiography sub-analysis due to a lack of stored echocardiographic loops or poor image quality. The overall cohort included 40 patients with a mean age of 65 ± 12 years at baseline, and 4 patients (10%) were female (Table 1). The mean body mass index was 28 ± 5 kg/m^2^, and 7 patients (18%) were diagnosed with diabetes mellitus prior to inclusion. According to randomization, 21 patients from the echocardiography sub-analysis were treated with ertugliflozin and 19 patients with placebo.

Patients had a mean LVEF of 40 ± 12%, a mean LV GLS of −13 ± −4%, and a mean left ventricular end-diastolic volume (LV EDV) of 195 ± 74 mL at baseline. The mean LAVI was 46 ± 19 mL/m^2^ at baseline. The mean right ventricle (RV) diameter was 38 ± 6 mm, the mean TAPSE was 20 ± 4 mm, and the mean inferior vena cava (IVC) was 15 ± 4 mm. Moderate or severe mitral regurgitation was present in 7 patients (18%) and moderate or severe tricuspid regurgitation in 10 patients (26%). There were no differences between treatment groups at baseline as illustrated in Table 1.

### 3.2. Changes in Cardiac Structure and Function After 52 Weeks Stratified by Treatment Group

Echocardiographic parameters remained unchanged after 52 weeks of treatment in both the ertugliflozin group and placebo group as illustrated in Table 2.

After adjustment for baseline values, there were no significant differences in change in structural and functional echocardiography parameters after 52 weeks of treatment with ertugliflozin versus placebo. An adjusted mean difference between treatment groups of −0.78 ± 1.20 in LVEF (*p* = 0.523), 5.19 ± 6.42 in LVEDV (*p* = 0.424), −1.83 ± 5.55 in left ventricular end-systolic volumes (LVESV) (*p* = 0.744), −2.54 ± 1.46 in right ventricle (RV) diameters (*p* = 0.091), and −0.10 ± 1.10 in TAPSE (*p* = 0.931) was calculated (Table 3). In a subgroup of 20 patients with adequate image quality to allow for myocardial deformation analysis, there was no significant difference in change in LV GLS between treatment groups (−0.44 ± 0.74, *p* = 0.567, Supplementary Table S1).

### 3.3. Association of Echocardiographic Parameters with Ventricular Arrhythmias

In a sub-analysis investigating associations between echocardiographic parameters and ventricular arrhythmias, lower LVEF at baseline was associated with a higher number of sVT/VF (Rate ratio [RR] 0.39, [CI 0.22, 0.71], *p* = 0.002) and nsVT (RR 0.50 [0.29, 0.84], *p* = 0.010) (Table 4). Lower TAPSE at baseline (RR 0.51 [0.27, 0.94], *p* = 0.031) was associated with more sVT/VF, but not with more nsVT (*p* = 0.941). Lower LVEDV was associated with more nsVT (0.47 [0.28, 0.80], *p* = 0.005), but not with sVT/VF (*p* = 0.2). There were no associations between LVESV and RV diameter and ventricular arrhythmias.

## 4. Discussion

In the echocardiography sub-analysis of the ERASe trial, in a total cohort of 40 patients (21 patients treated with ertugliflozin versus 19 patients treated with placebo), there were no significant differences in structural and functional echocardiographic parameters after 52 weeks of treatment.

As SGLT2is are now a class IA recommendation to treat all HF patients regardless of LVEF, the conduction of additional future RCTs investigating the effect of SGLT2is on ventricular arrhythmias or cardiac remodeling is highly unlikely in terms of ethical considerations [2]. To our knowledge, the ERASe trial is one of the last trials reporting results from a placebo-controlled trial about the effect of SGLT2is. The recently published EMPA-ICD trial showed a beneficial effect of empagliflozin compared to placebo in reducing ventricular arrhythmias in a cohort of 150 patients with type 2 diabetes with an ICD or CRT-D [21], which supports the findings from the ERASe trial. Thus, we believe the results of the ERASe echocardiography sub-analysis are important to report despite the limited sample size.

### 4.1. Structural and Functional Echocardiography Parameters and Ventricular Arrhythmias

Patients with severely reduced LVEF <35% have an increased risk of ventricular arrhythmias, leading to a class IA indication for the primary prevention ICD implantation in ischemic heart disease and IIa A recommendation in non-ischemic heart disease [1,22,23]. Our echocardiography analysis supports this evidence, showing an inverse association between LVEF and both sVT/VF and nsVT in a cohort of HF patients of both ischemic and non-ischemic entities treated with guideline-directed medical therapy (GDMT). Furthermore, our study showed worse right ventricular function, evaluated with TAPSE, to be associated with a higher amount of sVT/VF. Previous studies showed the additional prognostic value of right ventricular function on adverse clinical outcomes in HF patients on top of LVEF [24,25]. So far, studies showing the prognostic value of right ventricular function on ventricular arrhythmias in HF patients are rare. We hypothesize that reduced right ventricular function, in addition to decreased LVEF, is an indicator of advanced-stage disease and is thus associated with worse clinical outcomes including ventricular arrhythmias.

### 4.2. SGLT2is and Their Effect on Reverse Cardiac Remodeling

Adverse cardiac remodeling in HF patients and patients after myocardial infarction plays an important role in promoting disease progression, cardiac events, and mortality. Thus, treatment to reverse cardiac remodeling is crucial. GDMTs for HF, including angiotensin-converting-enzyme (ACE) inhibitors, angiotensin-receptor neprilysin-inhibitors (ARNIs), mineralocorticoid-receptor-antagonists (MRAs), and beta-blocker, have not only proven to reduce cardiovascular outcomes and mortality, but have shown to improve cardiac structure and function [26,27,28,29,30,31]. The potential effect of SGLT2is on reversal of cardiac remodeling is still not fully understood, and RCTs are scarce. A recently published Meta-Analysis from 2024 included six trials showing a beneficial effect of SGLT2is on LVEDV, LVESV, LVEF, and LAVI in HF patients with reduced ejection fraction [32]. The echocardiography sub-analysis of the EMMY trial supports this evidence gathered from HF trials in a post-myocardial infarction cohort, showing a significant effect on left ventricular volumes compared to a placebo after 26 weeks of empagliflozin, but it did not show improvement of LVEF [13]. The ERASe trial showed a significant reduction in ventricular arrhythmias after 52 weeks of treatment with ertugliflozin compared to placebo in a cohort of HF patients with non-preserved ejection fraction of both ischemic and non-ischemic etiologies. We speculated that the reduction in ventricular arrhythmias in the ertugliflozin group was driven by cardiac remodeling with a potential reduction in ventricular enlargement. This could not be confirmed in this echocardiography sub-analysis. Compared to the EMMY trial, the ERASe trial included patients of mixed etiologies not only focusing on ischemic heart disease, which could explain the diverging results of both echocardiography sub-analyses on cardiac remodeling. Additionally, the ERASe trial included patients at a more advanced disease stage when comparing baseline left ventricular volumes between the overall cohort of the ERASe trial versus the EMMY trial (LVEDV 169 mL [148, 245] vs. 122 mL [100, 142]) [13]. This might have influenced the ability to reverse cardiac remodeling with SGLT2i treatment in patients included in the ERASe trial.

### 4.3. SGLT2is and Their Impact on Sudden Cardiac Death (SCD)

As HF patients with impaired LVEF have an increased risk for ventricular arrhythmias and SCD, ICD implantation for primary prevention is part of the HF treatment regime. With the implementation of GDMT, cardiovascular outcomes and mortality in HF patients have been improved substantially over the last few decades [1,2]. Data on the SCD risk in HF patients after initiation of all four pillars of GDMT is scarce, especially in non-ischemic heart disease. Multimodality imaging including echocardiography and cardiac magnetic resonance (CMR) imaging helps in guiding SCD risk stratification in HF patients regardless of etiology [33,34]. As patients included in the ERASe trial suffered from a high arrhythmic burden, most patients were treated with an ICD as secondary prevention. Therefore, risk stratification in terms of primary prevention of SCD was not in the focus of the ERASe trial and data derived from CMR were not collected as they would have been beyond the scope of the trial. There is emerging evidence on the pleiotropic effects of SGLT2is beyond their already-known pharmacological profile. A recent meta-analysis by Matteucci et al. investigated the effect of empagliflozin and dapagliflozin on the risk reduction of SCD. In a pooled analysis of eight RCTs, treatment with SGLT2is showed a significant risk reduction of SCD [35]. This echocardiography sub-analysis from the ERASe trial supports the presumed reduction in SCD risk in patients treated with SGLT2is, independent of LVEF improvement. Future RCTs assessing the SCD risk of HF patients with impaired LVEF after initiation of GDMT to enable adequate risk stratification in those patients and avoid unnecessary ICD implantations with potential complications are still needed. Furthermore, the advances in device implantation with His-bundle pacing (HBP) and left bundle branch pacing (LBBP) are considered to offer a more physiological way of pacemaker stimulation compared to conventional CRT devices. So far there is only evidence from small studies not showing an improvement in cardiovascular mortality in patients treated with HBP or LBBP compared to CRT devices [36,37]. Larger trials are needed to show their effect on cardiovascular outcomes in HF patients including SCD.

### 4.4. Limitations

Due to the premature termination of the ERASe trial, the results of the echocardiography sub-analysis are based on a limited sample size, which diminishes the robustness of the findings. Thus, the results of this sub-analysis need to be interpreted with particular caution, as the study is underpowered and a type 2 error cannot be ruled out. Six patients were excluded from the analysis due to insufficient image quality. Although these patients were generally comparable to the overall study cohort with respect to baseline characteristics such as age, sex, body mass index, and diabetic status, a potential selection bias cannot be excluded for certain. Furthermore, assessment of myocardial deformation analysis was only possible in a limited number of patients due to a lack of adequate image quality. Additionally, with an inclusion of only 10% women in the ERASe trial, they are largely underrepresented and generalizability of our results to a female cohort is hardly feasible. The gender imbalance in HF trials is a common problem, as women are underrepresented in the majority of HF RCTs, with an average enrolment rate of 26% females in a systematic review from 2021 [38]. However, there are data, including a large meta-analysis of 26 different RCTs, indicating that GDMT is as effective in reducing cardiovascular outcomes in females despite their different baseline characteristics compared to men [39]. Nevertheless, data on adequate dosing and safety measurements in women are still scarce. Adaption of eligibility criteria for future HF trials to provide randomized controlled evidence from a large female cohort is urgently warranted.

## 5. Conclusions

Despite the significantly reduced primary endpoint of VT/VF burden in the ertugliflozin group of the ERASE trial, the echocardiography sub-analysis did not show improved structural and functional parameters assessed by echocardiography after 52 weeks of treatment compared to placebo. However, due to the premature termination of the ERASe trial, the small sample size may have influenced the results.

## Figures and Tables

**Table 1:** Comparison of baseline characteristics and echocardiographic parameters stratified by treatment group.

Baseline Characteristics	Overall Cohort (*n* = 40)	Ertugliflozin (*n* = 21)	Placebo (*n* = 19)	*p*-Value	*n* (% Miss)
Age, [years]	65 ± 12	65 ± 11	65 ± 13	0.964	40 (0%)
Female, *n* (%)	4 (10%)	2 (10%)	2 (11%)	>0.999	40 (0%)
Body mass index, [kg/m^2^]	28 ± 5	29 ± 5	27 ± 6	0.261	40 (0%)
Diabetes mellitus, *n* (%)	7 (18%)	3 (14%)	4 (21%)	0.689	40 (0%)
Echocardiographic parameters					
LV EDD, [mm]	60 ± 10	60 ± 8	60 ± 12	0.875	40 (0%)
LV ESD, [mm]	48 ± 10	47 ± 9	49 ± 11	0.658	37 (8%)
LV EDV, [mL]	195 ± 74	183 ± 59	207 ± 88	0.312	40 (0%)
LV ESV, [mL]	121 ± 64	108 ± 50	135 ± 74	0.187	40 (0%)
LVEF, [%]	40 ± 12	43 ± 10	37 ± 12	0.115	40 (0%)
LV GLS, [%]	−13 ± −4	−13 ± −3	−13 ± −4	0.835	20 (50%)
RV Diameter, [mm]	38 ± 6	37 ± 7	39 ± 4	0.302	39 (3%)
TAPSE, [mm]	20 ± 4	21 ± 4	19 ± 4	0.302	39 (3%)
LAVI, [mL/m^2^]	46 ± 19	47 ± 19	45 ± 21	0.759	29 (28%)
Moderate or severe AR, *n* (%)	1 (3%)	1 (5%)	0 (0%)	>0.999	38 (5%)
Moderate or severe MR, *n* (%)	7 (18%)	2 (10%)	5 (28%)	0.434	38 (5%)
Moderate or severe TR, *n* (%)	10 (26%)	3 (15%)	7 (39%)	0.323	38 (5%)
Tricuspid valve velocity, [m/s]	2.69 ± 0.54	2.64 ± 0.48	2.74 ± 0.60	0.650	24 (40%)
IVC Diameter, [mm]	15 ± 4	15 ± 3	16 ± 5	0.440	29 (28%)

All parameters are reported as absolute frequencies *n* (%) or means ± standard deviation. Abbreviations: AR—aortic regurgitation; IVC—inferior vena cava; LAVI—left-atrial volume index; LV EDD—left-ventricular end-diastolic diameter; LV EDV—left-ventricular end-diastolic volume; LVEF—left-ventricular ejection fraction; LV ESD—left-ventricular end-systolic diameter; LV ESV—left-ventricular end-systolic volume; LV GLS—left-ventricular global longitudinal strain; MR—mitral regurgitation; RV—right ventricle; TAPSE—tricuspid annular plane systolic excursion; TR—tricuspid regurgitation.

**Table 2:** Echocardiographic parameters at visit 1 and visit 2 stratified by treatment group.

	Ertugliflozin		Placebo	
	Baseline *n* = 21	Week 52 *n* = 21	Baseline *n* = 19	Week 52 *n* = 19
LV EDD, [mm]	60 ± 8	60 ± 10	60 ± 12	60 ± 10
LV ESD, [mm]	47 ± 9	48 ± 9	49 ± 11	48 ± 10
LV EDV, [mL]	183 ± 59	184 ± 67	207 ± 88	208 ± 91
LV ESV, [mL]	108 ± 50	102 ± 45	135 ± 74	135 ± 78
LVEF, [%]	43 ± 10	44 ± 9	37 ± 12	38 ± 12
LV GLS, [%]	−13 ± −3	−14 ± −2	−13 ± −4	−13 ± −5
RV Diameter, [mm]	37 ± 7	35 ± 5	39 ± 4	39 ± 6
TAPSE, [mm]	21 ± 4	20 ± 4	19 ± 4	19 ± 5
Moderate or severe AR, *n* (%)	1 (5%)	1 (6%)	0 (0%)	0 (0%)
Moderate or severe MR, *n* (%)	2 (10%)	1 (6%)	5 (28%)	4 (24%)
Moderate or severe TR, *n* (%)	3 (15%)	1 (6%)	7 (39%)	5 (29%)
Tricuspid valve velocity, [m/s]	2.64 ± 0.48	2.56 ± 0.27	2.74 ± 0.60	2.48 ± 0.48
IVC diameter, [mm]	15 ± 3	16 ± 4	16 ± 5	15 ± 4

All parameters are reported as absolute frequencies *n* (%) or means ± standard deviation. Abbreviations: AR—aortic regurgitation; IVC—inferior vena cava; LV EDD—left-ventricular end-diastolic diameter; LV EDV—left-ventricular end-diastolic volume; LVEF—left-ventricular ejection fraction; LV ESD—left-ventricular end-systolic diameter; LV ESV—left-ventricular end-systolic volume; LV GLS—left-ventricular global longitudinal strain; MR—mitral regurgitation; RV—right ventricular; TAPSE—tricuspid annular plane systolic excursion; TR—tricuspid regurgitation.

**Table 3:** Estimates for differences in echocardiographic parameters between treatment groups from linear regression model after adjustment for baseline values.

Echocardiographic Parameters	Baseline	Follow-Up	Adjusted Difference	*p*-Value
	Mean ± SD	Adjusted Mean ± SD	Mean ± SD	
LVEF [%]				
Placebo	37.21 ± 2.83	41.50 ± 0.84	−0.78 ±1.20	0.523
Ertugliflozin	43.05 ± 2.25	40.80 ± 0.82		
LV EDV [mL]				
Placebo	207.47 ± 20.28	193.00 ± 4.56	5.19 ±6.42	0.424
Ertugliflozin	182.81 ± 12.87	198.00 ± 4.43		
LV ESV [mL]				
Placebo	135.11 ± 17.09	119.00 ± 3.92	−1.83 ± 5.55	0.744
Ertugliflozin	107.76 ± 10.94	117.00 ± 3.81		
RV Diameter [mm]				
Placebo	38.74 ± 0.90	38.3 ± 1.02	−2.54 ± 1.46	0.091
Ertugliflozin	36.85 ± 1.55	35.8 ± 1.02		
TAPSE [mm]				
Placebo	19.42 ± 0.94	19.70 ± 0.77	−0.10 ± 1.10	0.931
Ertugliflozin	20.80 ± 0.92	19.60 ± 0.77		

All parameters are reported as means ± standard deviation. Abbreviations: LV EDV—left-ventricular end-diastolic volume; LVEF—left-ventricular ejection fraction; LV ESV—left-ventricular end-systolic volume; RV—right ventricular; SD—standard deviation; TAPSE—tricuspid annular plane systolic excursion.

**Table 4:** Associations between echocardiographic parameters at baseline and ventricular arrhythmias.

Echocardiographic Parameters	Rate Ratio	95% CI	*p*-Value
sVT/VF			
LVEF (1-SD change)	0.39	0.22, 0.71	0.002
LV EDV (1-SD change)	0.66	0.36, 1.23	0.2
LV ESV (1-SD change)	1.1	0.59, 2.06	0.8
RV Diameter (1-SD change)	1.04	0.56, 1.93	0.9
TAPSE (1-SD change)	0.51	0.27, 0.94	0.031
nsVT			
LVEF (1-SD change)	0.5	0.29, 0.84	0.01
LV EDV (1-SD change)	0.47	0.28, 0.80	0.005
LV ESV (1-SD change)	0.68	0.39, 1.17	0.2
RV Diameter (1-SD change)	1.31	0.74, 2.30	0.4
TAPSE (1-SD change)	1.02	0.59, 1.78	0.941

Abbreviations: LV EDV—left-ventricular end-diastolic volume; LVEF—left-ventricular ejection fraction, LV ESV—left-ventricular end-systolic volume; nsVT—non-sustained ventricular tachycardia; RV—right ventricular; SD—standard deviation; sVT—sustained ventricular tachycardia; TAPSE—tricuspid annular plane systolic excursion; VF—ventricular fibrillation.

## Data Availability

The data underlying this article will be shared on reasonable request to the corresponding author.

## Supplementary Materials

The following supporting information can be downloaded at: https://www.mdpi.com/article/10.3390/jcm14238294/s1, Table S1: Estimates for differences in LV GLS between treatment groups from linear regression model after adjustment for baseline values.

## Abbreviations

The following abbreviations are used in this manuscript:

LVEF	Left ventricular ejection fraction
RCT	Randomized controlled trial
ICD	Implantable cardioverter-defibrillator
CRT-D	Cardiac resynchronization therapy-device
SGLT2i	Sodium–glucose co-transporter 2 inhibitor
