# Supplementary material for: Impact of Ertugliflozin on Cardiac Structure and Function in Patients with ICDs/CRT-Ds Assessed by Echocardiography: A Post Hoc Sub-Analysis of the ERASe Trial
Source: J Clin Med. 2025 Nov 22;14(23):8294. doi: 10.3390/jcm14238294 (PMC12693279; doi:10.3390/jcm14238294)
Supplement: Supplementary file 1 [file jcm-14-08294-s001.zip › jcm-3948860-supplementary.pdf]

Supplementary Table S1. Estimates for differences in LV GLS between treatment groups from linear regression model after adjustment for baseline values.

Echocardiographic parameters	Baseline (N = 20)	Follow-up (N = 17)	Adjusted difference	P-value
	**Mean ± SD**	**Adjusted Mean ± SD**	**Mean ± SD**	
**LV GLS**				
Placebo	13.04 ± 1.36	13.80 ± 0.49	-0.44 ±0.74	0.567
Ertugliflozin	13.40 ± 1.03	13.40 ± 0.55		

All parameters are reported as means ± standard deviation. Abbreviations: LV GLS left ventricular global longitudinal strain; SD standard deviation.
